# USP21 regulates Hippo pathway activity by mediating MARK protein turnover

**DOI:** 10.18632/oncotarget.19322

**Published:** 2017-07-18

**Authors:** Hung Thanh Nguyen, Jan-Michael Kugler, Anand C. Loya, Stephen M. Cohen

**Affiliations:** ^1^ Department of Cellular and Molecular Medicine, University of Copenhagen, Copenhagen, Denmark; ^2^ Department of Pathology, Rigshospitalet, Copenhagen, Denmark

**Keywords:** Hippo pathway, MARK, LATS, YAP, ubiquitin

## Abstract

The Hippo pathway, which acts to repress the activity of YAP and TAZ trancriptional co-activators, serve as a barrier for oncogenic transformation. Unlike other oncoproteins, YAP and TAZ are rarely activated by mutations or amplified in cancer. However, elevated YAP/TAZ activity is frequently observed in cancer and often correlates with worse survival. The activity and stability of Hippo pathway components, including YAP/TAZ, AMOT and LATS1/2, are regulated by ubiquitin-mediated protein degradation. Aberrant expression of ubiquitin ligase complexes that regulate the turnover of Hippo components and deubiquitylating enzymes that counteract these ubiquitin ligases have been implicated in human cancer. Here we identify the USP21 deubiquitylating enzyme as a novel regulator of Hippo pathway activity. We provide evidence that USP21 regulates YAP/TAZ activity by controlling the stability of MARK kinases, which promote Hippo signaling. Low expression of USP21 in early stage renal clear cell carcinoma suggests that USP21 may be a useful biomarker.

## INTRODUCTION

The Hippo pathway has emerged as a major barrier for oncogenic transformation [1]. Hippo signaling represses the activity of the transcriptional co-activators YAP and TAZ, so that loss of pathway activity leads to increased YAP/TAZ activity. Previous studies have shown that expression or activity of Hippo pathway components are frequently suppressed in cancer, similar to other key tumor suppressor pathways such as PTEN, p14^ARF^/p53 and p16/pRb (reviewed in [2]). NF2, a promoter of Hippo signaling is the Hippo pathway component most often inactivated by mutation, as reported in the COSMIC database (reviewed in [2]) and its loss is associated with a heritable cancer syndrome [3]. Inactivation of other Hippo genes such as RASSF1A, FAT1-4, RASSF2, RASSF4 through mutation or silencing was also observed in cancer (reviewed in [2, 4]). Similarly, increased YAP/TAZ expression or an increase in their activity has frequently been documented in comprehensive surveys of solid-tumor cancers, including lung, colorectal, pancreatic, hepatocellular, ovarian, and prostate carcinomas [5–8]. Tumors with high YAP activity often correlate with worse cancer survival [9–12]. Interestingly, elevated YAP or TAZ activity also confers tumor cells with the ability to evade anti-cancer drugs. Using synthetic lethality screens or RNAi approaches, two groups have recently reported that activated YAP made B-Raf mutant or Ras-harboring tumor cells resistant to MEK-targeted cancer therapies [13, 14]. Together, these studies have shed light on the importance of Hippo deregulation in cancer development and suggest that the Hippo pathway may play a key role in the acquisition of drug resistance. Further studies on the mechanisms of Hippo pathway regulation and its deregulation in cancer will be important in suggesting new avenues of therapeutic approach.

Several lines of evidence suggest that regulation of Hippo pathway proteins through ubiquitin-mediated degradation plays an important role in human cancer. The core Hippo pathway kinases, mammalian sterile-20-like (MST1/2) and large tumor suppressor kinase 1/2 (LATS1/2), phosphorylate the YAP and Taz proteins and prevent them from exerting their roles as transcriptional co-activators that promote carcinogenesis. Phosphorylated YAP and TAZ are targeted by the bTrCP/SCF ubiquitin ligase system for degradation [15, 16]. YAP protein turnover is regulated by Ras signaling, through regulation of SOCS5/6 expression, which recruits YAP to an Elongin B/C-Cullin5 ubiquitin ligase complex [17]. While promoting the degradation of YAP and Taz is central to the effect of activated Hippo signaling, the ubiquitin-mediated degradation process has been shown to occur at multiple nodes in the Hippo pathway to regulate YAP/Taz activity. The E3 ligase PRAJA2 promotes degradation of MOB1, an upstream regulator of LATS kinases, and has been shown to promote glioblastoma [18]. The NEDD4 family of E3-ligase proteins have been shown to regulate the abundance of LATS kinases, AMOT and AMOT-like proteins, which suppress YAP activity [19–21]. As a consequence, an increased expression of these E3 ligases is sufficient to promote tumorigenesis [22]. We recently reported that deubiquitylating (DUB) enzyme USP9X could act as a tumor suppressor by promoting the stability of AMOT proteins and suppressing YAP/TAZ activity. USP9X is under-expressed in many kidney tumors, where its expression level correlated with patient survival [23]. Another deubiquitylating enzyme, DUB3, was also reported to regulate YAP/TAZ activity by mediating the stability of multiple Hippo components, such as ITCH, LATS kinases and AMOT proteins [24].

Here we present evidence that the deubiquitylating enzyme USP21 controls YAP/TAZ activity indirectly, by regulating the stability of the MARK family protein kinases. USP21 deubiquitylates MARK proteins to control their stability. The MARK kinases in turn regulate LATS1/2 kinase activity and thereby regulate YAP/Taz phosphorylation and stability. We provide evidence that USP21 limits anchorage-independent growth of transformed primary cells and cancer cell lines. USP21 protein was expressed at low levels in a majority of renal clear cell carcinoma (RCC) samples, suggesting that low USP21 activity could increase cancer relevant cellular phenotypes.

## RESULTS

### USP21 mediates YAP activity

**Figure 1:**
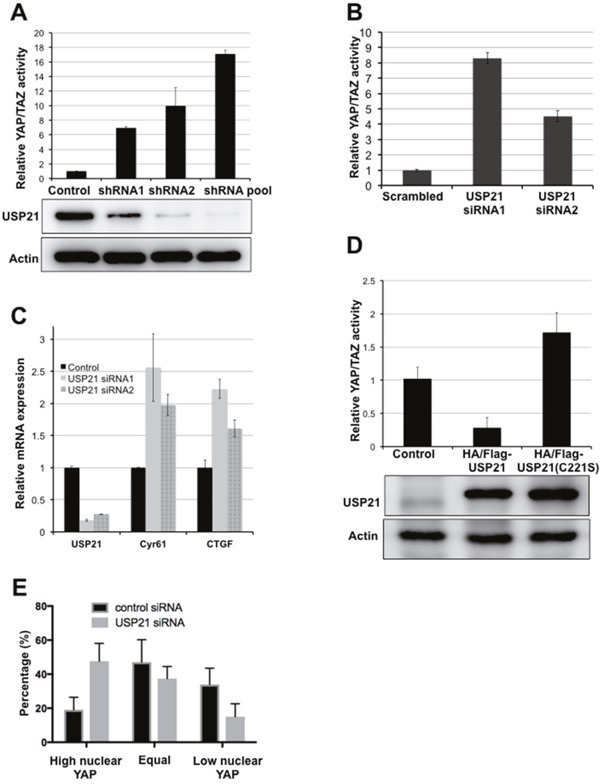
USP21 regulates YAP/TAZ activity **(A)** Luciferase reporter assays showing USP21 shRNAs increases YAP/TAZ activity. HEK293T cells were co-transfected to express the YAP/TAZ reporters together with USP21 shRNAs or with a control vector. YAP/TAZ activity was calculated as a ratio of firefly to Renilla activity. Data were normalized to the relevant control and represent the mean of three independent transfection experiments ± SD. **(B)** Luciferase reporter assays showing YAP/TAZ activity. HEK293T cells were co-transfected with reporter plasmids together with USP21 siRNAs or with a control siRNA. Data represent the mean of three independent transfection experiments ± SD. **(C)** Quantitative PCR for the expression of YAP transcriptional targets. HEK293T cells were transfected to express a scrambled control or independent siRNAs targeting USP21. Data represent the average of 3 independent experiments ± SD. **(D)** Luciferase reporter assays showing YAP/TAZ activity. HEK293T cells were co-transfected to express the reporters together with a plasmid expressing USP21 wild type or mutant (C221S) or a control vector. Data represent the mean of three independent replicates ± SD. **(E)** YAP localization was scored as previously described [24]. Representative images are provided in Supplementary Figure 2. Data represent the average of three independent experiments ± SD.

USP21 was identified in a shRNA screen for DUBs that regulate YAP/TAZ activity (ref [23]; the HGNC designation for this protein has been changed from USP23 to USP21). Depletion of USP21 by two independent shRNAs or a pooled shRNA mixture significantly increased YAP reporter activity (Figure 1A) in HEK293T cells. These shRNAs efficiently reduced USP21 protein expression (Figure 1A). The effect of USP21 depletion was reproduced by using two independent siRNAs targeting USP21 (Figure 1B). These siRNAs strongly reduced USP21 mRNA levels, measured by qPCR (Figure 1C), and led to a significant increase in expression of the YAP transcriptional targets, CTGF and Cyr61 (Figure 1C). We observed a similar effect of USP21 depletion in several other cell types. Stable expression of USP21 shRNAs led to increased CTGF and Cyr61 expression in BJ fibroblasts, lung carcinoma A549 cells and in metastatic breast cancer MDA-MB-231 cells (Supplementary Figure 1).

Overexpression of USP21 had the opposite effect, suppressing YAP reporter activity (Figure 1D). Expression of a catalytically inactive mutant form of USP21C221S [25] showed no inhibitory effect on YAP reporter activity (Figure 1D), although the USP21^C221S^ mutant protein was expressed at a comparable level to the native protein. Instead, the USP21^C221S^ mutant appeared to increase YAP reporter activity, perhaps suggesting a dominant negative effect; though we note that the effect was not strong enough to be statistically significant (p=0.07). These results suggest that the enzymatic activity of USP21 is required for its inhibitory effect on YAP activity.

Nuclear localization of YAP is often taken as an indicator of increased YAP activity. Nuclear localization of YAP increased significantly in BJ cells depleted of USP21, compared to the control cells (Figure 1E, Supplementary Figure 2), consistent with the observation that USP21 inhibition increased YAP reporter activity and transcriptional expression of YAP target genes. Taken together, these data indicate that USP21 regulates the transcriptional activity of YAP.

### USP21 regulates MARK kinase stability

**Figure 2:**
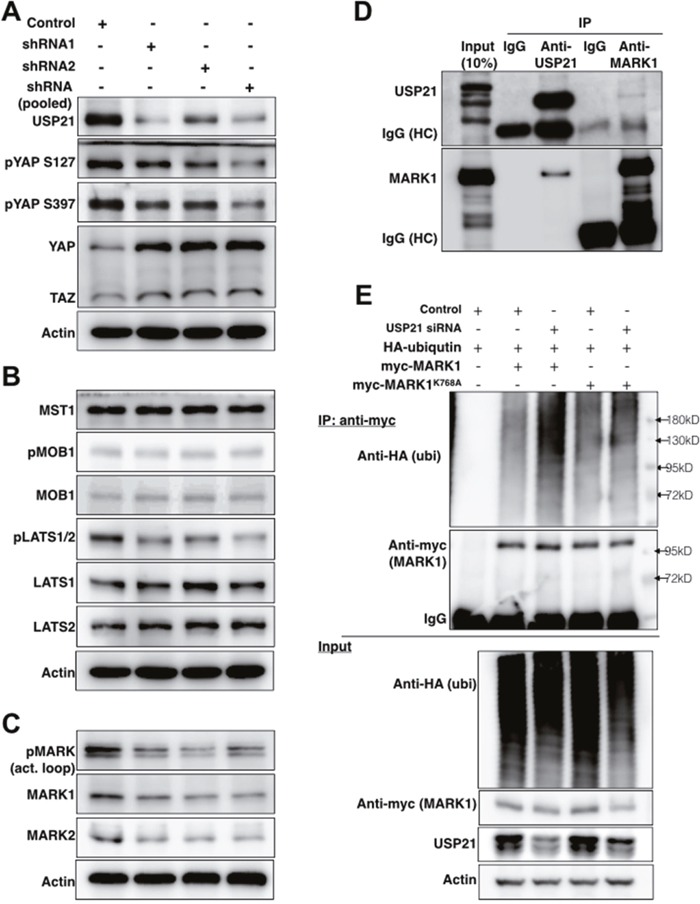
USP21 regulates the stability of MARK proteins and MARK1 ubiquitylation **(A-C)** Immunoblots showing effects of USP21 depletion on Hippo pathway components in HEK293T cells. Blots were probed with the indicated antibodies. Anti-Actin was used to control for loading. Data in panels A-C are from the same blots, so the same anti-Actin controls were used. **(D)** Immunoblots showing co-immunoprecipitation of MARK1 and USP21. Lysates from MG132-treated HEK293T cells were immunoprecipitated with agarose-conjugated antibodies against USP21 or MARK1 or IgG control antibodies overnight. Separate blots were probed with antibodies against USP21 and MARK1. **(E)** Immunoblots of HEK293T cells co-transfected to express myc-MARK1 (or myc-MARK1K768A)/HA-tagged ubiquitin and a scrambled or USP21-specific siRNA to deplete USP21 for 36h. Lysates were immunoprecipitated with anti-myc to recover MARK1 and blots were probed with anti-HA (for ubiquitin detection), anti-myc (MARK1), anti-USP21 and anti-Actin.

To examine how USP21 regulates YAP/TAZ activity, we examined the effects of USP21 depletion on the expression levels of Hippo pathway proteins. HEK293T cells were transfected to express shRNAs independently or in a pool targeting USP21. There was a strong increase in total protein levels of YAP and to a lesser extent of TAZ (Figure 2A). This was accompanied by reduced YAP phosphorylation at Ser127 and S397 (Figure 2A), which are target sites of LATS kinases. Given that phosphorylation of these sites targets YAP and TAZ for degradation, the effect of USP21 depletion on YAP levels is likely a consequence of the reduced YAP turnover. Reduced phosphorylation could be due to reduced expression or activity of the LATS kinases. However, USP21 depletion did not change the level of LATS1 or LATS2 proteins. Instead, phosphorylation of LATS1/2 (Thr1079/1041) was strongly reduced (Figure 2B), indicating reduced LATS kinase activity. This suggested that the effects of USP21 might be mediated via a kinase that phosphorylates the LATS proteins.

We next tested the effect of USP21 depletion on known regulators of LATS kinases, MST kinases and the LATS1/2 coactivator MOB1 (reviewed in [26]). Depletion of USP21 did not significantly change the phosphorylation of MOB1 or the level of total MOB1 protein (Figure 2B). Total MST1 was also unaffected (Figure 2B, phosphorylated MST1/2 was not detected unless the cells were treated with phosphatase inhibitors, and so could not be assessed).

Microtubule affinity-regulating kinase (MARK) family proteins are serine/threonine kinases that were recently shown to serve as regulators of LATS1/2 kinases [27]. Importantly, in this context the MARK kinases act on LATS proteins independently of the MST kinases [27]. The MARK kinase family consists of 4 closely related proteins encoded by different loci [27, 28]. USP21 depletion reduced the expression of MARK1 and 2 proteins (Figure 2C), but not the mRNA transcripts (Supplementary Figure 3A). USP21 depletion also led to reduced phosphorylation of the MARK activation loop. This may be due to the reduced level of MARK proteins in these cells. Conversely, overexpression of USP21, but not of the catalytically inactive C221S mutant, stabilized MARK1 and MARK2 protein expression (Supplementary Figure 3B). These observations indicate that USP21 regulates the stability of the MARK family proteins.

To ask which of the MARK isoforms are involved in regulation of YAP activity, we depleted the MARK kinase family members individually and measured YAP reporter activity in HEK293T cells. qPCR analysis confirmed that the siRNAs selectively depleted their target transcripts in an efficient manner (Supplementary Figure 4A). Depletion of MARK3 had little or no effect on YAP reporter activity; MARK1 and 4 depletion increased YAP activity 2-5 fold; and depletion of MARK2 caused over a 20 fold increase (Supplementary Figure 4B). Next, we tested the effects of USP21 depletion is cells co-depleted for MARK kinases using siRNAs specific to individual MARK kinases. Reducing MARK1, 2 levels attenuated the effect of USP21 depletion on YAP activity, while MARK4 depletion has a more limited effect (Supplementary Figure 5). These observations provide evidence that all three MARK kinases contribute to mediating the effects of USP21 on YAP/TAZ activity.

All of the MARK protein isoforms have been shown to interact with USP21 in assays using overexpressed epitope-tagged proteins [29, 30]. We sought to examine whether USP21 could interact with MARK1 at endogenous levels. MARK1 was previously identified as a possible target for ubiquitylation [31], and so was expected to be unstable if USP21 was involved in deubiquitylating MARK proteins. For this reason, we treated HEK 293T cells with MG132 to limit proteasome-mediated protein degradation prior to preparing lysates for immunoprecipitation (IP). Under these conditions, we observed co-IP of MARK1 when performing IP with antibody to USP21 (Figure 2D). Reciprocally, USP21 was recovered in the anti-MARK1 IP (Figure 2D). These assays provide evidence for interaction of the endogenous proteins.

Next, we asked whether USP21 affects the ubiquitylation status of MARK1. As shown in Figure 2E, USP21 depletion increased the amount of ubiquitin detected on MARK1 purified by IP. The peptide sequence SGTSIAFKNIASKIA from MARK1 containing lysine 768 has been identified as a ubiquitylated substrate [31]. The K768A mutant form of MARK1 showed considerably less ubiquitylation following USP21 depletion than the wild type MARK1 protein (Figure 2E), suggesting that K768 is a site at which USP21 dequbiquitylates MARK1. Overexpression of USP21 significantly reduced ubiquitin incorporation into MARK1 but has less impact on the MARK1K768A mutant (Supplementary Figure 6). These findings suggest that USP21-mediated deubiquitylation regulates the stability of MARK1.

### USP21 depletion enhances oncogenic transformation

**Figure 3:**
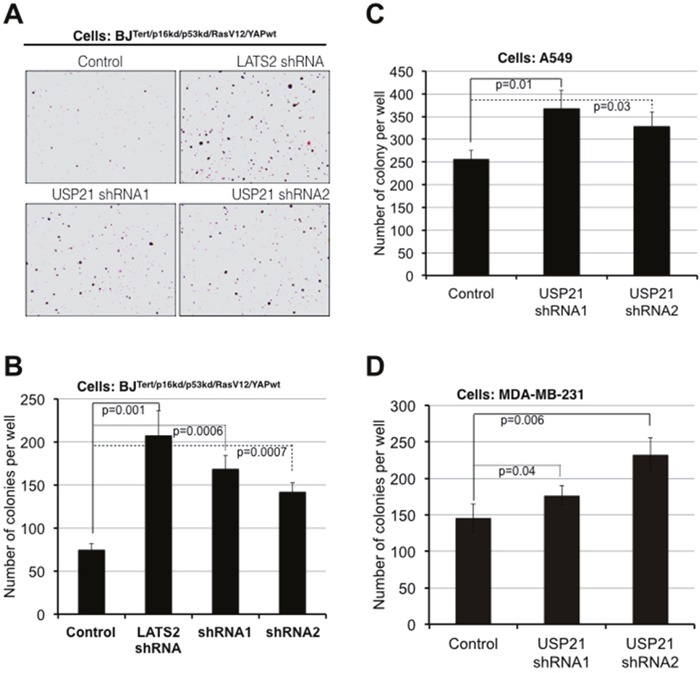
USP21 inhibition promotes oncogenic transformation in BJ and cancer cell lines Assays for colony formation in soft agar. **(A, B)** BJT/p53kd/p16kd/RasG12V/YAP^wt^cells, **(C)** A549 lung cancer cells, **(D)** MDA-MB-231 breast cancer cells. Cells were transduced to express shRNAs against USP21 or a control. After antibiotic selection, cells were plated in soft agar for 10 days (A549 cells), 2 weeks (BJ cells) and 3 weeks (MDA-MB-231 cells). Representative images of colonies formed from BJ cells after 2 weeks **(A)**. Average colony number per well ± SD from three independent experiments of BJ **(B)**, A549 **(C)** and MDA-MB-231 **(D)** cells. *p* values were determined using the student's t test (2-tailed, unequal variance).

**Figure 4:**
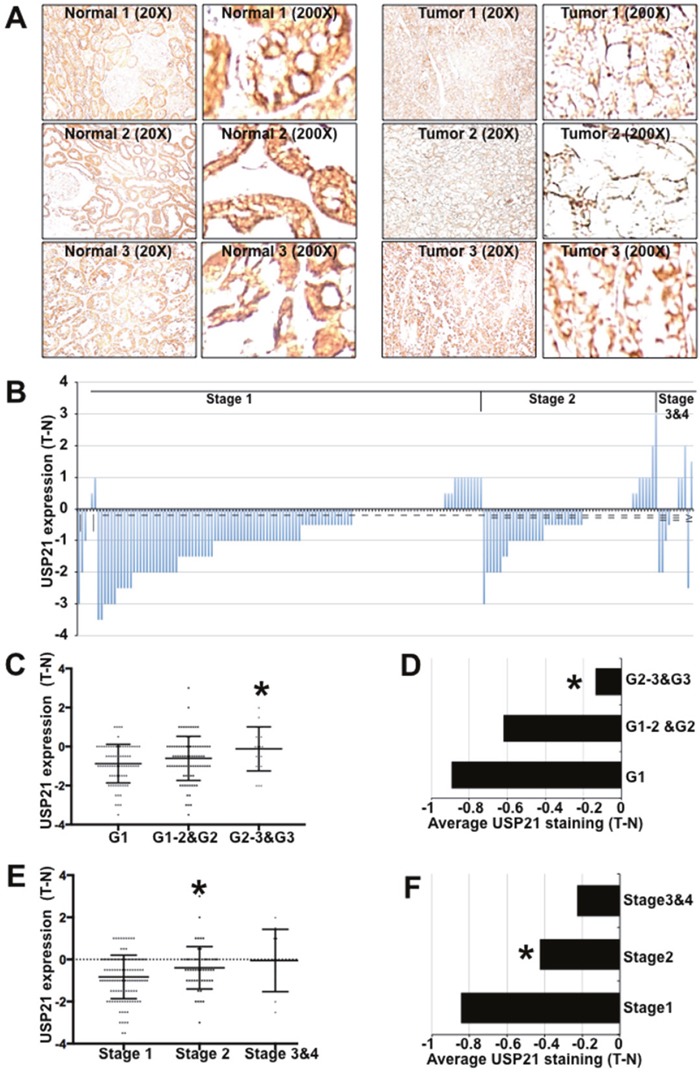
USP21 expression in kidney tumors and adjacent matched normal tissues **(A)** Immunohistochemical staining of kidney tissues (in pairs of tumor and adjacent normal samples) with anti-USP21. **(B)** Plot showing histopathology scores for anti-USP21 staining of RCC tumors and matched adjacent normal tissue. The slides were examined by experienced pathologists to confirm the tissue identity and assigned a score: 0 (no staining), 1 (weak staining of less than 10% of tissue), 2 (weak staining of 10–25% of tissue), 3 (weak to moderate staining of up to 50% tissue), 4 (moderate to strong staining of 50–75% of tissue), 5 (moderate to strong staining of more than 75% of tissue). Data show the IHC score for the tumor minus that of adjacent normal tissue. **(C, E)** IHC scores for anti-USP21 staining of individual RCC samples separated by histological grade. Data show the average of the tumor-normal scores. * indicates a statistically significant difference using the Mann-Whitney test (p=0.006 in C and = 0.02 in E). **(D, F)** Histopathology scores for anti-USP21 staining of RCC samples separated by tumor stage. Data show the average of the tumor-normal scores. Student's t-test (2-tailed, unequal variance) was used to determine the significance of differences among groups. * indicates p=0.002 **(D)** and =0.01 **(F)**.

Our findings thus far have shown that USP21 limits YAP/TAZ activity, through regulation of MARK kinase turnover. Given the importance of YAP/TAZ activity in cellular transformation, we wanted to ask whether USP21 might limit transformation through regulation of YAP/TAZ. To test this, we employed an assay based on anchorage-independent growth of partially transformed primary human fibroblasts (BJ fibroblast cells expressing shRNAs to deplete p53/p16 and activated Ras^G12V^ along with native YAP in place of the small t oncoprotein that is conventionally used along with Ras^G12V^ to transform primary cells to anchorage independence) [1]. We previously found that small t could be replaced by expression of a moderate level of native YAP protein, and that the amount of soft-agar colony formation in these assays was very sensitive to the amount of YAP activity [1]. As a control, we verified that depletion of LATS2 kinase strongly increased soft agar colony formation in this experiment (Figure 3A and 3B). Expression of two independent shRNAs targeting USP21 also significantly increased colony formation (Figure 3A and 3B). USP21 depletion also led to increased soft agar colony formation in A549 and MDA-MB-231 cancer cell lines (Figures 3C and 4D: recall that YAP target gene expression was increased in these cancer cell lines following USP21 depletion, Supplementary Figure 1). Thus, depletion of USP21 can increase YAP/TAZ activity and increase the ability of cells to grow in an anchorage independent manner. These findings suggest that USP21 could have tumor suppressive activity, mediated through regulation of YAP/TAZ.

### USP21 expression in human cancer

Next, we sought to investigate the expression of USP21 protein in human cancer. An initial survey of data from ProteinAtlas.org suggests that USP21 protein is abundant in human uterine, gastric and kidney normal tissues, detected by immunohistochemical staining. Interestingly, USP21 expression was lost or reduced in all 12 reported cases of renal clear carcinomas (RCC), compared to adjacent normal tissues (http://www.proteinatlas.org/ENSG00000143258-USP21/cancer).

Tissue microarrays provided access to a larger collection of RCC samples paired with normal kidney tissue from the same patients. We performed immunohistochemical labeling to investigate the protein expression of USP21 (Figure 4A) using the tested USP21 antibody (HPA028397) recommended by the Protein Atlas. In normal tissue, renal tubules and glomerulus cells exhibited moderate to strong cytoplasmic immuno- reactivity. USP21 expression was lost or reduced in 116 tumors (∼62%), unchanged in 45 cases (24%) and increased in 25 cases (14%) (Figure 4B). Surprisingly, we noted that the intensity of USP21 protein down-regulation was greater in lower stage and lower grade tumors and that this difference was smaller in more advance stage/grade tumors (Figure 4C-4F).

## DISCUSSION

Suppression of Hippo signaling results in increased YAP activity, which can contribute to oncogenic transformation in many types of human cancer. This study provides evidence that USP21 acts through stabilization of MARK kinases to limit YAP/TAZ activity. Loss of USP21 expression would therefore lead to reduced MARK-Hippo signaling resulting in increased expression of oncogenic YAP. The normal activity of USP21 may contribute to tumor suppression.

Consistent with this mechanism, we found that expression of USP21 protein is reduced or lost in a majority of human RCC tumors, compared to paired normal tissue from the same individual. We did not observe a significant correlation between USP21 protein expression and overall cancer survival, for the 89 paired samples with patient survival information. This might be a consequence of the sample size. However, we noted that USP21 is located in region 1q21 of chromosome 1, which is frequently amplified in human cancers. This region contains many oncogenes such as CREB3L4 and MDM2 and increase in their copy number has been linked with worse survival for many human cancers [32–35]. The fact that USP21 is located in this amplified region may have obscured any association between USP21 expression with cancer survival. Indeed, we observed that the intensity of USP21 protein down-regulation became less profound in more advanced cancers, suggesting that selection for regional amplification might lead to a secondary increase in USP21 concomitant with disease progression. It is tempting to speculate that low USP21 may contribute to tumor formation early in cancer development, but that this effect might be obscured by secondary genetic changes including amplification of region 1q21.

USP21 may regulate different signaling pathways and its effect on oncogenic transformation may depend on the prevailing signaling that controls cell growth. A previous study showed that USP21 deubiquitylates receptor-interacting protein 1, a suppressor of TNF-induced NF-KB activation. Thus, USP21 can act as a negative regulator of NF-KB signaling [36]. Another study showed that USP21 expression is upregulated in bladder tumors and suggested that USP21 could promote cancer growth and metastasis by inhibiting the ubiquitylation of EZH2 in bladder cancer cell lines [37]. In this study, the small subset of RCC tumors (14%) displayed an increased expression of USP21 as compared to their matched normal tissues and the diminishment of downregulated USP21 in higher grade or more advanced tumors might suggest that additional signaling regulated by USP21 contribute to cancer progress. In addition, we also found that USP21 inhibition suppressed soft agar growth of metastatic breast cancer MDA-MB-468 cell lines (Supplementary Figure 7). These apparently opposite effects of USP21 in different cancers and cell lines suggest that the impact of USP21 depletion may depend on the underlying pattern of genetic and epigenetic alterations found in different tumors and cellular contexts.

## MATERIALS AND METHODS

### Antibodies

Antibodies to MARK2 (#9118), phospho-MARK activation loop (#4836) YAP/TAZ (#8418), pYAP S127 (#4911), pYAP S397 (#13619), LATS1 (#9153), LATS2 (#13646), pLATS1/2 Thr1079/1041 (#8654), MOB1 (#3863), phosphor-MOB1 (Thr35, #8699), MST1 (#3682), phosho-MST1/2 (Thr183/180, #3681)) and Myc Tag (#2278) were from Cell Signaling Technology (Danvers, MA, USA). HA antibody (#sc-7392) was from Santa Cruz Biotechnology (Dallas, TX, USA). Anti-USP21, anti-MARK1, anti-actin, HA-, and Myc-conjugated beads were from Sigma-Aldrich (St Louis, MO, USA).

### Plasmids, siRNAs and shRNAs

8xGTIIC-luciferase was a gift from Stefano Piccolo (Addgene plasmid #34615). The pRL-CMV (Renilla, #E2261) was purchased from Promega (Madison, WI, USA). HA/Flag-USP21 was a gift from Wade Harper (Addgene plasmid # 22574). The pBabe-myc-MARK1 expression plasmid was cloned by PCR using cDNA from HEK293T cells. Flag-USP21, Flag-USP21^C221S^ and myc-MARK1^K768A^ mutants were generated by PCR as previously described [38] and subcloned into pBabe expression vector. The HA-ubiquitin expression plasmid was a kind gift from Dr. Simon Bekker-Jensen (University of Copenhagen). LATS2 shRNA was described in [39]. USP21 siRNAs were purchased from Sigma-Aldrich. Details of the shRNAs sequences used to deplete USP21 are provided in Supplmentary Table 1.

### Luciferase assays

Luciferase assay to measure YAP/TAZ activity were performed as described (ref) using a dual luciferase kit (E1960, Promega).

### Quantitative real-time RT-PCR

RNA extraction, cDNA synthesis and quantitative PCR were performed as previously described [23]. qPCR primer sequences are provided in Supplmentary Table 1.

### Cell culture, transfection, immunoblotting, immunoprecipitation and ubiquitin assay

BJ fibroblast, A549, MDA-MB-231, MDA-MB-468 and HEK293T cells were obtained from ATCC and cultured in DMEM (Sigma) with 10% fetal calf serum (HyClone) and 1% penicillin-streptomycin. HEK293T cells were transfected with using the Calcium phosphate method. Western blotting was performed as previously described [23]. For co-immunoprecipitation, HEK293T cells were pretreated overnight with 5μM MG132, washed once with cold PBS and lysed with PLC buffer [40] containing 50 mM Hepes pH 7.5, 150 mM NaCl, 5 % Glycerol, 0.5 % Triton X-100, 1.5 mM MgCl2, 1 mM EGTA supplemented with 20 μg/ml RNAase A, 1 mM DTT, 1 mM Na_3_VO_4_ and protease inhibitor cocktail on ice for 30 minutes. Supernatant was collected after centrifugation and pre-cleared with proteins A/G beads (Santa Cruz, sc#2003). Antibodies against isotype controls, USP21 and MARK1 were pre-incubated with proteins A/G agarose for 2h before being immunoprecipitated with pre-cleared cell lysates overnight at 4°C. Agarose beads were washed 4x with PLC buffer before being subjected to Western blot analysis. For ubiquitin assays, a mixture of 5μg of myc-MARK1 expression plasmid and 2μg of HA-ubiquitin expression plasmid was co-transfected with either scrambled control or USP21 siRNA (final concentration of 50 nM) in 10-cm disc of HEK293T cells. 36h-transfected cells were further treated with MG132 5μM overnight before being harvested for immunoprecipitation. Cells were washed once with cold PBS lysed in the PLC buffer freshly supplemented with 20mM of N-ethylmaleimide (NEM, Sigma). Pre-cleared cell lysate was incubated with myc antibody-conjugated agarose on a rotator at 4°C for 3h. Beads were washed 4x with lysis buffer and eluted as recommended by the manufacturer.

### Immunohistochemical analysis

Tissue arrays (KD1503, KD1504 and HKid-CRC180Sur-01) containing 186 pairs of kidney clear cell carcinoma and their matched normal tissues were obtained from the US Biomax, Rockville, MD, USA. Immunochemical staining of tissue arrays was performed and samples were analyzed by 2 experienced pathologists as previously described [23].

### Viral transduction and soft agar assay

Amphotropic retroviruses were made as described previously [41]. Supernatant from transfected Phoenix-Ampho cells (Nordic Biosite ApS, Copenhagen, Denmark) was harvested at 36–48 h, snap-frozen in liquid nitrogen and stored in aliquots at −80°C for subsequent viral transductions. Cells were plated to reach 70% confluence when infected with viruses overnight in the presence of 8 μg/ml of polybrene. Antibiotic selection was started after 36h of infection. Stable cells were plated for soft agar growth assay as described [1].

### Statistical analyses

The Student's t-test (2-tailed, unequal variance) was used to determine the significance of differences among conditions for luciferase assays and quantitative PCR. The Chi-square test was used to assess the significances of differences in subcellular localization of YAP.

## SUPPLEMENTARY MATERIALS FIGURES AND TABLE



## Abbreviations

DUB: (deubiquitylating enzymes)
YAP: (yes-Associated Protein)
TAZ: (tafazzin)
LATS: (large tumor suppressor)
MARK: (microtubule affinity regulating kinase)
bTrCP/SCF: (beta-transducin repeat containing/Skp
Culin and: F-box containing complex)
COSMIC: (catalogue of somatic mutations in cancer)
RCC: (renal clear cell carcinoma)
