# Supplementary material for: USP21 regulates Hippo pathway activity by mediating MARK protein turnover
Source: Oncotarget. 2017 Jul 18;8(38):64095–105. doi: 10.18632/oncotarget.19322 (PMC5609986; doi:10.18632/oncotarget.19322)
Supplement: Supplementary file 1 [file oncotarget-08-64095-s001.pdf]

USP21 regulates Hippo pathway activity by mediating MARK protein turnover

SUPPLEMENTARY MATERIALS

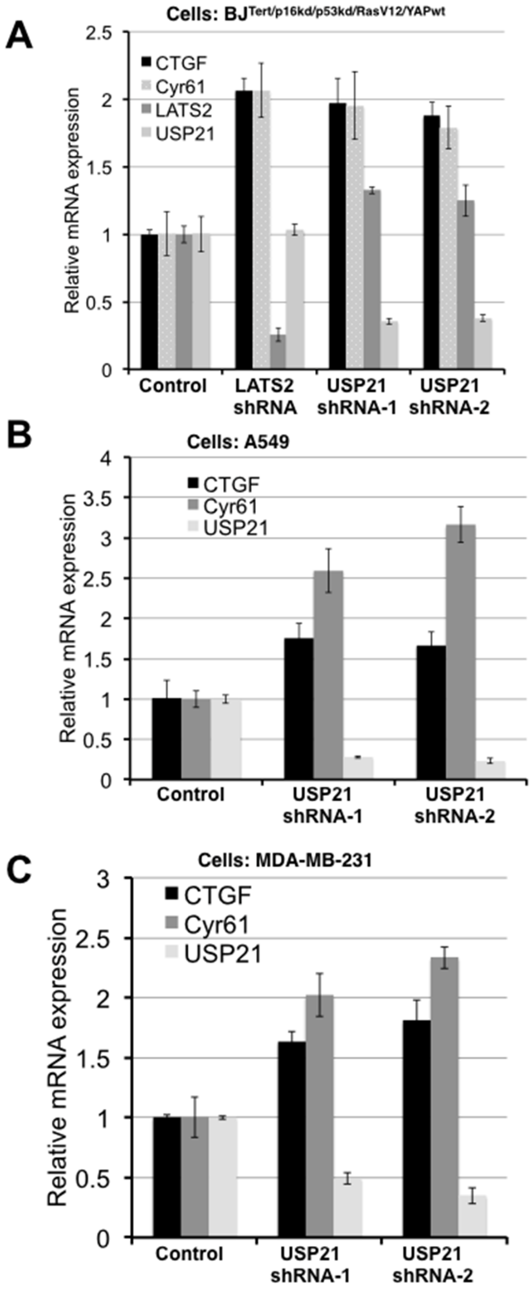

Supplementary Figure 1: USP21 inhibition upregulates transcriptional expression of YAP target genes. Quantitative PCR was used to measure YAP target transcript levels in foreskin fibroblast BJ$^{Tert/p16kd/p53kd/RasV12/YAPwt}$ **(A)**, lung cancer A549 **(B)** and breast cancer MDA-MB-231 **(C)** cell lines were transduced and selected to stably express the indicated shRNAs. shRNA-mediated depletion of LATS2 was used as a control for the effect of increasing YAP/TAZ activity. GAPDH mRNA level was used for normalization. RNA levels for USP21 and LATS2 are shown to monitor knockdown efficiency. CTGF and Cyr61 are transcriptional targets of YAP and their mRNA expression was used as a measure of YAP activity. Data represent the average of three independent experiments ± SD.

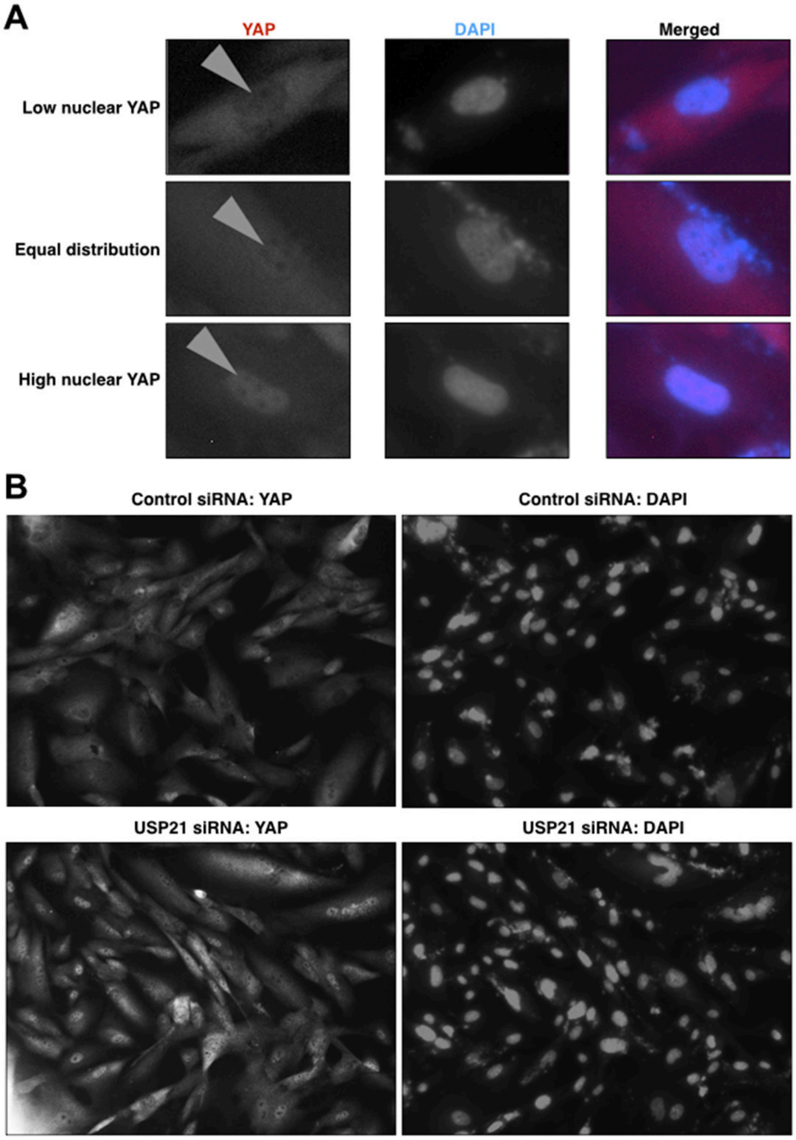

Supplementary Figure 2: Effect of USP21 knockdown on YAP localization. Cells were seeded for 24h before transfection with USP21-specific siRNAs or scrambled controls. Cells were fixed after 36h and stained with YAP antibody. qPCR was used to confirm the depletion of USP21. **(A)** Representative images showing subcellular localization of YAP protein. We visually compared the intensity of YAP label in nucleus and cytoplasm and assigned each cell a score: low nuclear YAP, equal distribution or high nuclear YAP. **(B)** Representative images showing YAP labeling in fields of cells treated with control or USP21 siRNAs.

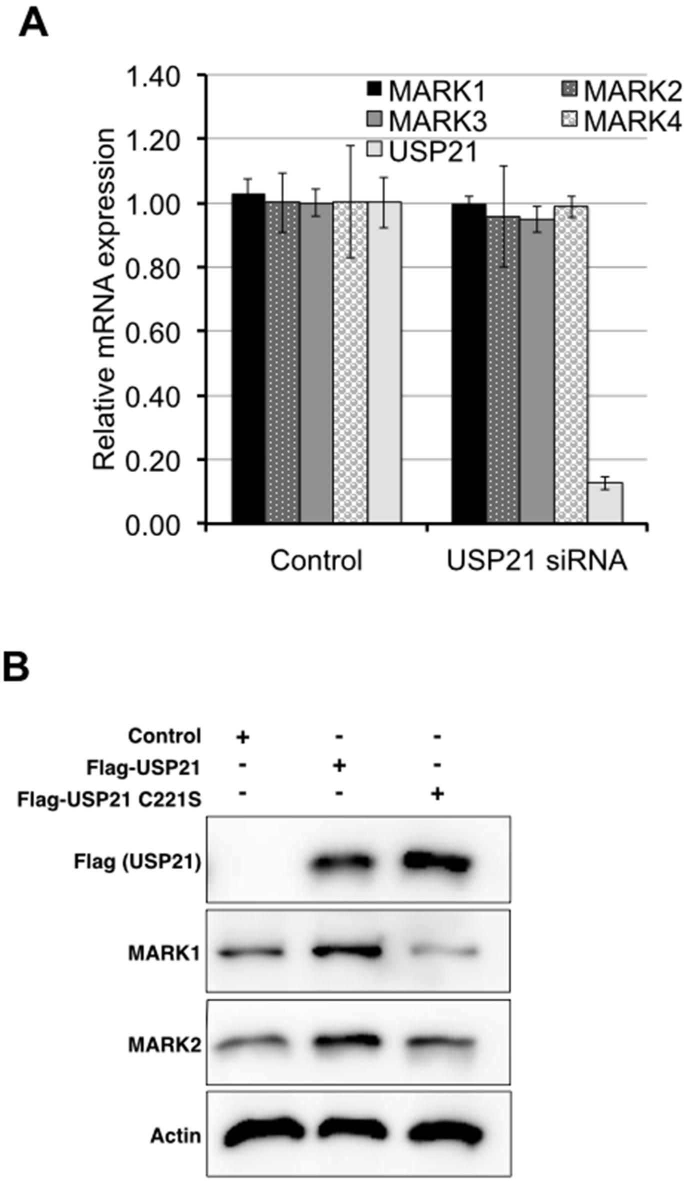

Supplementary Figure 3: Effect of USP21 depletion on MARK mRNA expression. **(A)** Quantitative PCR was used to measure transcript levels of MARK1, 2, 3 and 4 in 293T cells treated with control or USP21 siRNAs. GAPDH mRNA was used for normalization. **(B)** Immunoblots showing effects of USP21 or USP21^{C221S} overexpression on MARK1 and MARK2 proteins in HEK293T cells. Blots were probed with the indicated antibodies. Anti-Actin was used to control for loading.

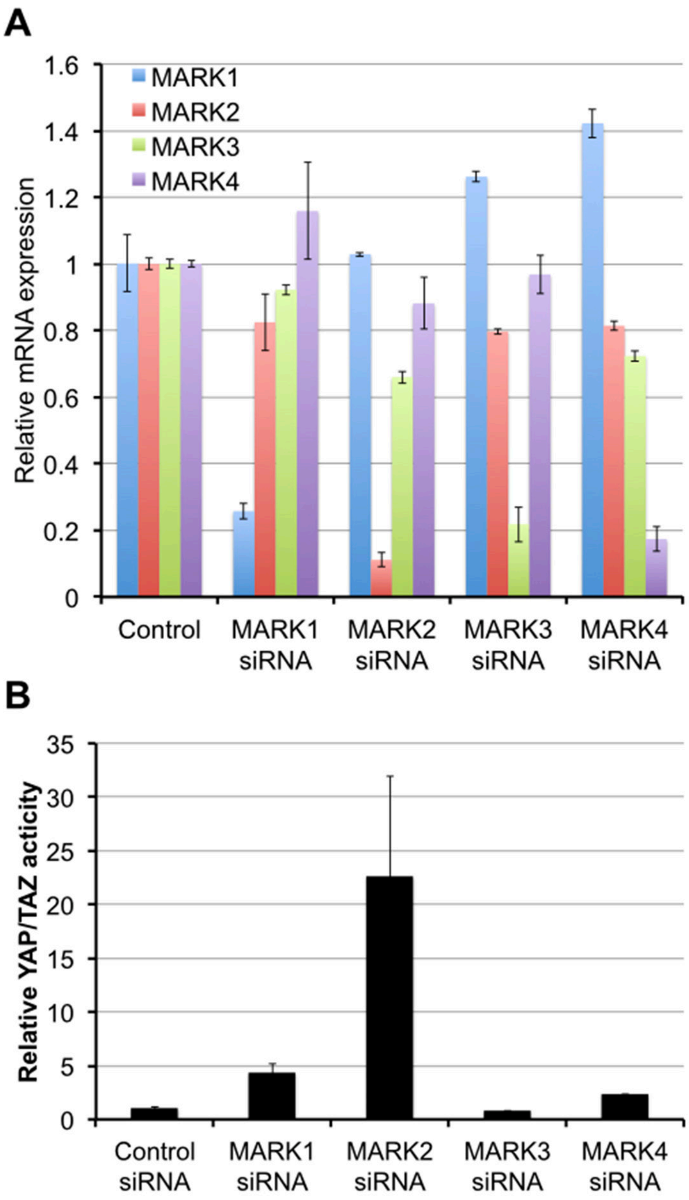

Supplementary Figure 4: Effect of MARK protein inhibition on YAP activity. **(A)** Luciferase reporter assays showing the effect of individual siRNAs against MARK proteins on YAP/TAZ activity. HEK293T cells were co-transfected to express the reporters together with specific siRNAs against each MARK gene or with a control vector. Firefly and Renilla activities were measured 48h after transfection. Data were normalized to the relevant control and represent the mean of three independent transfection experiments ± SD. **(B)** Quantitative PCR of mRNA MARK transcripts. The same HEK293T cell samples, which were co-transfected to express the reporters together with specific siRNAs against MARK proteins or with a control vector for luciferase assay, were used for RNA extraction. GAPDH mRNA level was used for normalization. mRNA expression of MARK1, 2, 3 and 4 were measured to assess the siRNA efficiency. Data represent the average of three independent experiments ± s.d.

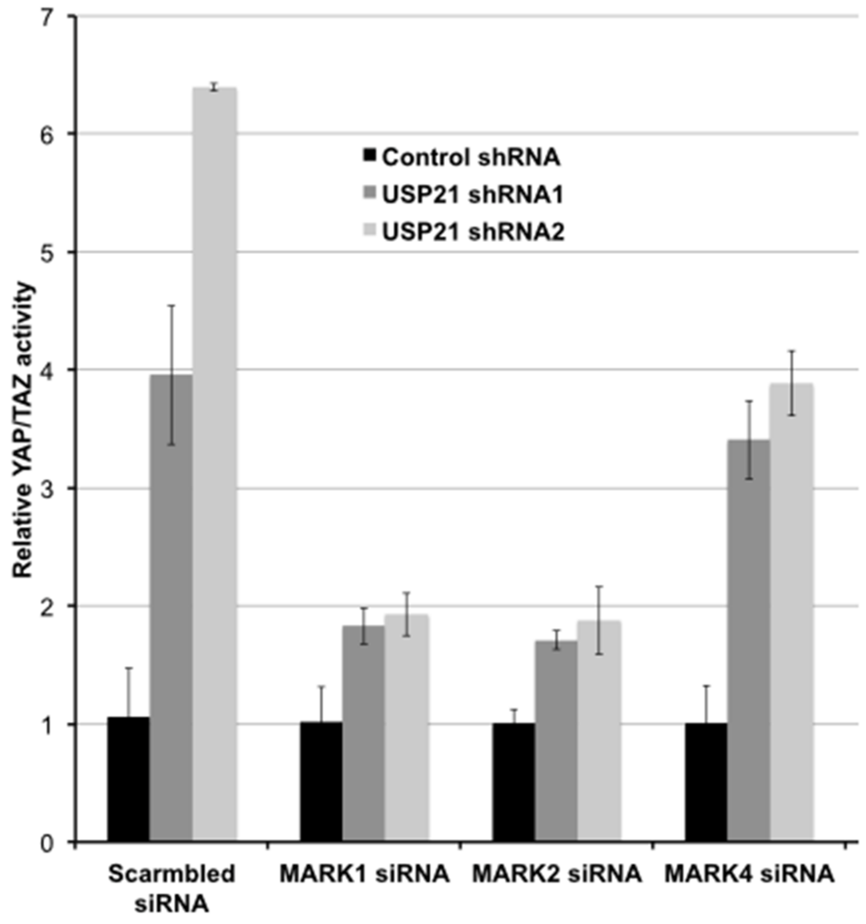

Supplementary Figure 5: Effect of USP21 shRNAs on YAP activity in MARK depleted cells. Luciferase reporter assays showing the effect of individual USP21 shRNAs on YAP/TAZ activity in cells treated with control or MARK1, 2, 4 siRNAs. HEK293T cells were co-transfected to express a mixture of the reporters and siRNA targeting MARK1, 2, 4 or control siRNA, together with specific USP21 shRNAs or a control shRNA. Firefly and Renilla luciferase activities were measured 48h after transfection. The effect of USP21 shRNAs was normalized with the control for each group. The data represent the mean of three independent transfection experiments ± SD.

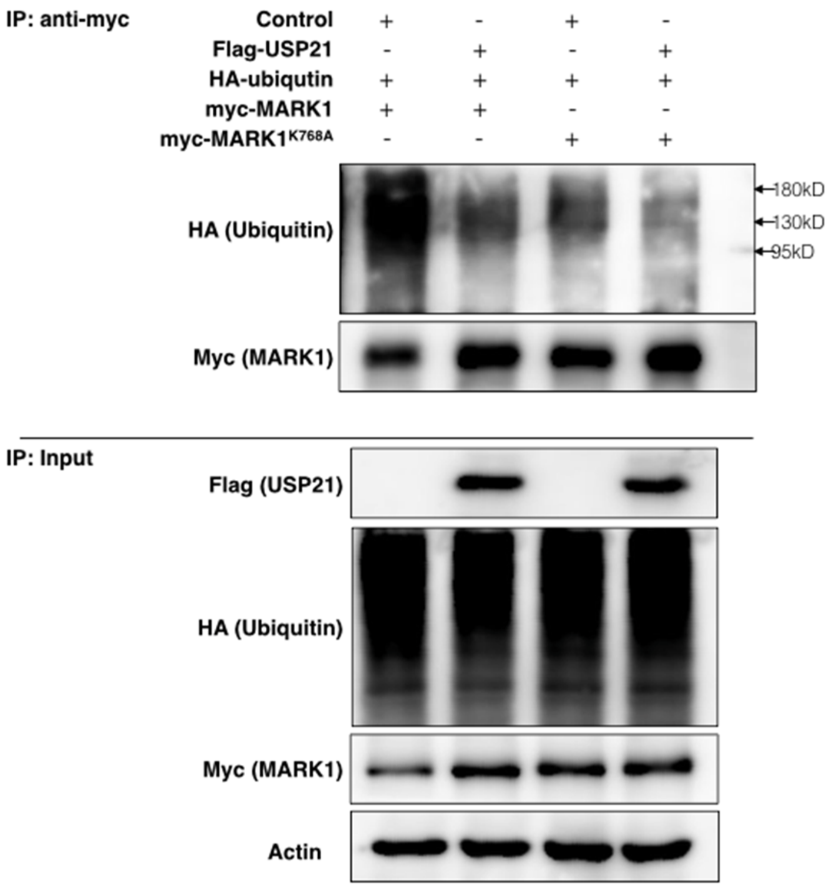

Supplementary Figure 6: Effect of USP21 overexpression on ubiquitylation of MARK1 and MARK1K768A. Immunoblots of HEK293T cells co-transfected to express myc-MARK1 (or myc-MARK1^{K768A})/HA-tagged ubiquitin and a control or Flag-USP21-expressing plasmid for 36h. Lysates were immunoprecipitated with anti-myc to recover MARK1 and blots were probed with anti-HA to visualize HA-Ubiquitin, anti-myc (MARK1), anti-Flag and anti-Actin.

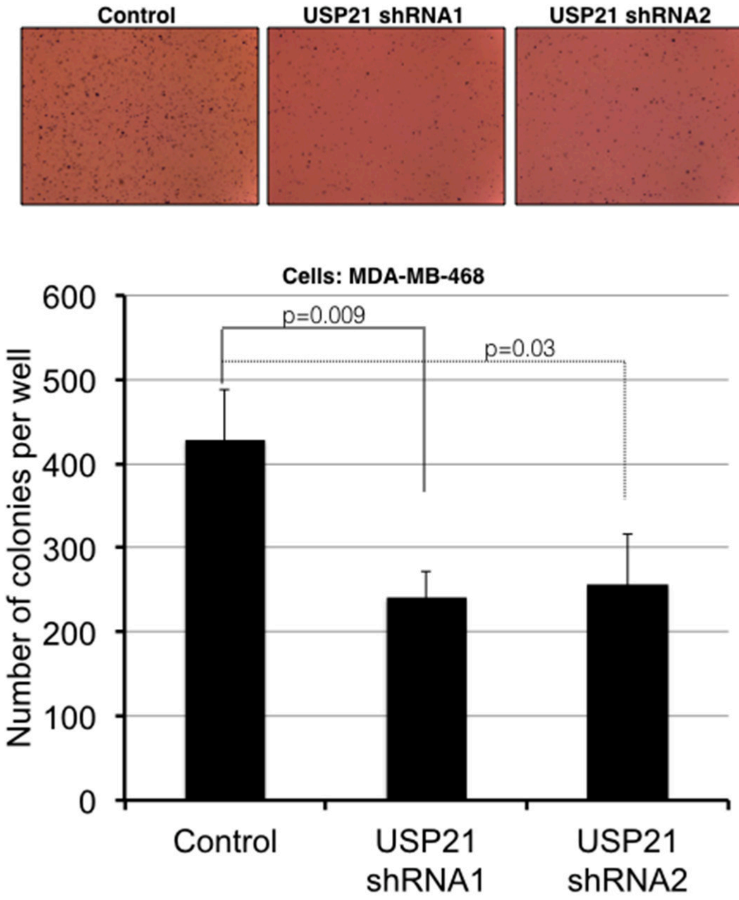

Supplementary Figure 7: USP21 knockdown inhibits colony formation of MDA-MB-468 cells. MDA-MB-468 cells were transduced to express independent shRNAs against USP21 or a control vector. After antibiotic selection, cells were plated in soft agar. *Up*: representative images of colonies formed after 3 weeks. *down*: average colony number per well ± SD from three independent experiments. *p* values were determined using the student's t test (2-tailed, unequal variance).

Supplmentary Table 1: shRNA target sequences and primers used for quatitation PCR

Gene	Sequence
USP21 shRNA1	Target sequence: GGTGTCTCTGCGGGATTGT
USP21 shRNA2	Target sequence: AGTTCAGTAGGTGTAGACT
MARK1 (qPCR)	Forward: GAGTTTCTACACCGGGGGAT
	Reverse: GAGCGGGACACGGAAAAT
MARK2 (qPCR)	Forward: CACATTGGAAACTACCGGCTC
	Reverse: GGAGGAGTTCAGTTGAGTCTTGT
MARK3 (qPCR)	Forward: TGGATTCAACTGAGTTTTGTCA
	Reverse: TGTTGAAAACAATCGGCAAG
MARK4 (qPCR)	Forward: TGAAGGGCCTAAACCACCC
	Reverse: CCAGCACTTGCGTACTCCA
USP21 (qPCR)	Forward : GGTAGCTTGGATCCCACTCG
	Reverse: GCCTCACTGGGGGACAG
GAPDH (qPCR)	Forward : ACATCAAGAAGGTGGTGAAGCAG
	Reverse: CAAAGGTGGAGGAGTGGGTGTC
Cyr61 (qPCR)	Forward: TATTCACAGGGTCTGCCCTC
	Reverse: AACGAGGACTGCAGCAAAA
CTGF (qPCR)	Forward: TAGGCTTGGAGATTTTGGGA
	Reverse: GGTTACCAATGACAACGCCT
